# Supplementary material for: metaMIC: reference-free misassembly identification and correction of de novo metagenomic assemblies
Source: Genome Biol. 2022 Nov 14;23:242. doi: 10.1186/s13059-022-02810-y (PMC9661791; doi:10.1186/s13059-022-02810-y)
Supplement: Supplementary file 1 — Additional file 1: Fig. S1-S29. [file 13059_2022_2810_MOESM1_ESM.pdf]

# metaMIC: reference-free Misassembly Identification and Correction of *de novo* metagenomic assemblies

## *Supplementary materials*

Senying Lai, Shaojun Pan, Chuqing Sun, Luis Pedro Coelho, Wei-Hua Chen, Xing-Ming Zhao

# Supplementary methods and results

## Insert size estimation

Insert size estimation was performed only on the read pairs satisfying the following conditions:

1) Both reads are mapped to the same contig
2) The read pairs have correct orientations

For a paired-end read $r(r_l, r_r)$, the insert size is calculated as follows:

$$Insert\_size = P_{re} - P_{ls} + 1, \text{ where}$$

$P_{ls}$: the position of left mate read $r_l$ starting at the contig

$P_{re}$: the position of right mate read $r_r$ ending at the contig

The expected insert size ($\mu$) was calculated as the median value of all insert sizes, whereas the standard deviation ($\sigma$) was estimated by the median absolute deviation of insert sizes:

$$\sigma = k \cdot median(|Insert\_size_i - \mu|), \qquad where\ k = (1/\Phi^{-1}(3/4)) \approx 1.4826$$

## Contig-based features

The contig features applied by metaMIC were categorized into four feature types: 1) read pair consistency; 2) read coverage; 3) nucleotide variants; 4) $k$-mer abundance difference (KAD). Each feature type consists of several features and are explained in detail below.

1. ***Read pair consistency***

Each read is assigned to the following six categories if satisfying the conditions:

Proper read: 1) paired-end reads with left and right reads mapping to the same contig; 2) the insert size belongs to $[\mu - 3\sigma, \mu + 3\sigma]$. 3) The read pairs have correct orientations.

Discordant read with wrong insert size (Type 1 discordant read): 1) paired-end reads with left and mate reads mapping to the same contig; 2) the insert size does not belong to $[\mu - 3\sigma, \mu + 3\sigma]$. 3) The read pairs have correct orientations.

Discordant read with incorrect orientation (Type 2 discordant read): 1) paired-end reads with left and mate reads mapping to the same contig; 2) The read pairs have incorrect

orientations.

Discordant read with different mapping locations (Type 3 discordant read): paired-end reads with left and mate reads mapping to different contigs.

Clipped read: the read contains at least 20 unaligned bases at either end of the read.

Supplementary read: different parts of the read are aligned to different regions of contigs.

metaMIC will count the number of above six read types, and all sub-features belonging to the read pair consistency are listed as below:

| **Read pair consistency** | |
|---|---|
| Name | Description |
| proper_read_ratio | The proportion of proper read among all reads mapping to the contig |
| discordant_size_ratio | The proportion of type 1 discordant read among all reads mapping to the contig |
| inversion_read_ratio | The proportion of type 2 discordant read among all reads mapping to the contig |
| discordant_loc_ratio | The proportion of type 3 discordant read among all reads mapping to the contig |
| clipped_read_ratio | The proportion of clipped read among all reads mapping to the contig |
| supplementary_read_ratio | The proportion of supplementary read among all reads mapping to the contig |

### *2. Read coverage*

Coverage-based statistics including read coverage and fragment coverage are calculated at each base of the assembly through using the information from the input of BAM file. Read coverage is the number of reads which are mapped over that base at a given base of an assembly. For fragment coverage, fragments are considered instead of reads, and a fragment is defined as the region between the outermost ends of a proper read pair. Fragment coverage is the number of fragments that are mapped to that position given a base in an assembly. Then both read coverage and fragment coverage at each position $i$ of contig $c$ are further standardized by the following formulas, and $L_c$ is the length of the contig c.

$$Standardized\_read\_coverage_{ic} = \frac{read\_coverage_{ic}}{\left(\sum_{j=1}^{L_c} read\_coverage_{jc}\right)/L_c} ,$$

$$Standardized\_fragment\_coverage_{ic} = \frac{fragment\_coverage_{ic}}{\left(\sum_{j=1}^{L_c} fragment\_coverage_{jc}\right)/L_c} ,$$

Standardized deviations of read coverage and fragment coverage for a given contig *c* are calculated by the formulas:

$$\sigma_{read\_coverage_c} = \sqrt{\frac{\sum_{j=1}^{L_c}(standardized\_raad\_coverage_{jc} - 1)^2}{L_c}} ,$$

$$\sigma_{fragment\_coverage_c} = \sqrt{\frac{\sum_{j=1}^{L_c}(standardized\_fragment\_coverage_{jc} - 1)^2}{L_c}} .$$

As the reads mapped to a perfect assembled contig are mostly the proper read pairs, we expected the read coverage to be more closer to the fragment coverage at a given base of an assembly. thus we calculated the difference between the fragment coverage and read for a given contig *c* as follows:

$$Cov\_diff_{ic} = standardized\_raad\_coverage_{ic} - standardized\_fragment\_coverage_{ic}$$

$$Cov\_diff_c = \left(\sum_{j=1}^{L_c} Cov\_diff_{jc}\right)/L_c$$

All sub-features of each contig belonging to the read coverage are listed as below:

| **Read coverage** | |
|---|---|
| Name | Description |
| normalized_cov_dev | $\sigma_{read\_coverage_c}$ of contig *c* |
| normalized_frag_cov_dev | $\sigma_{fragment\_coverage_c}$ of a contig *c* |
| abnormal_cov_width | The proportion of bases in a contig c with $Standardized\_read\_coveArge_{ic} \notin [0.5,1.5]$ |
| abnormal_frag_cov_width | The proportion of bases in a contig c with $Standardized\_fragment\_coveArge_{ic} \notin [0.5,1.5]$ |
| cov_diff | $Cov\_diff_c$ of contig *c* |

### *3. Nucleotide variants*

Nucleotide variants information at each position of a given assembly are extracted from the pileup output of the BAM file. For each position *i* of an assembly, the read bases in reads mapped to that position were categorized into following types if satisfying conditions:

Matched read base: the mapped read base is consistent with the base at position *i* in the contig.

Mismatched read base: the mapped read base is disagreement with the base at position *i* in

the contig.

Deleted read base: There is a deletion after this mapped read base.

Inserted read base: There is an insertion after this mapped read base.

Ambiguous read base: The mapped read base in the read is an ambiguous base (N)

The proportion of each type of read base mapped to a given contig is calculated, and all sub-features belonging to nucleotide variants are listed below:

| **Nucleotide variants** | |
|---|---|
| Name | Description |
| correct_portion | The number of matched read bases across a contig divided by the total number of read bases mapped to that contig |
| disagree_ portion | The number of mismatched read bases across a contig divided by the total number of read bases mapped to that contig |
| deletion_ portion | The number of deleted read bases across a contig divided by the total number of read bases mapped to that contig |
| ambiguous_ portion | The number of ambiguous read bases across a contig divided by the total number of read bases mapped to that contig |
| insert_ portion | The number of inserted read bases across a contig divided by the total number of read bases mapped to that contig |

***4. k-mer abundance difference***

*k*-mer abundance different (KAD) is calculated for a given *k*-mer in a contig using the following formula:

$$KAD\ value = log_2\frac{(c+m)}{m(n+1)}, where$$

$n$: copies of the *k*-mer in the contig.

$c$: copies of the *k*-mer in the reads mapped to the contig

$m$: the occurrence of single-copy *k*-mers of the contig in the mapped reads (also regarded as the sequencing depth of the contig)

a *k*-mer with $KAD \notin [-0.5, +0.5]$ is regarded as an error *k*-mer. For each base in a contig, a base will be regarded as an error base if it is the start of an error *k*-mer, and the KAD value of the base is assigned according to the KAD value of that *k*-mer. All the sub-features belonging to this category are listed below:

| *k*-mer abundance difference | |
|---|---|
| Name | Description |
| mean_KAD | Average KAD value of bases across a contig |
| KAD_dev | Standardized deviation of KAD value over bases across a contig |
| abnormal_KAD_ratio | Proportion of error bases across the contig |

## Window-based features

For window-based features, four different types of features mentioned above were calculated for a window region instead of a contig. In more detail, the number of different types of read pairs, read bases as well as error bases are counted within each window region, and then further standardized by the length of window region or the number of mapped reads/read bases within each region. The standardized deviation of both coverages are also calculated within each window region.

## Relative importance of input features

To quantify the contribution of individual input features on misassembly predictions, we analyzed the relative importance of contig-based features for each assembler-specific random forest model. We found that the top important features mainly belong to the read pair consistency based features for both IDBA_UD and MEGAHIT assembled contigs although the top ranked features are slightly different for each assembler specific model. The proportion of discordant reads with their mates mapped to contigs (discordant_loc_ratio) was ranked first in MEGAHIT specific model, while the proportion of proper reads (proper_read_ratio) was the top first important feature in IDBA_UD specific model. The difference between top features of assembler specific models may be explained by the different distribution of misassembly types in the contigs generated by different assemblers. For instance, inter-genome translocation generally causes discordant read pairs with different mapped locations, and it was also indeed the most common assembly error generated by MEGAHIT (see Figure S6).

## Thresholds of anomaly score and read breakpoint ratio setting for detecting error regions in isolate genomes

For exploring how different thresholds of anomaly score used for detecting error regions in isolate gnomes will influence the performance, we set different thresholds ranging from 0.8 to 1, and evaluated the results on four isolate genomic assemblies from GAGE-B dataset. Then the number of error regions with anomaly score higher than the selected threshold were regarded as true positives (TP), whereas the total number of window regions that contain no misassembly breakpoints with anomaly score higher than the threshold corresponds to the false positives (FP). The false negative (FN) is the number of error regions with anomaly score below the threshold. Therefore, we calculated the precision, recall and corresponding F1 scores at different anomaly score threshold settings on all isolate genomic assemblies, and the threshold of read breakpoint ratio was set 0.2 based on the histogram of read breakpoint ratios over erroneous and error-free regions (Fig. S20). As shown in Figure S24, a lower anomaly score can identify more misassemblies, however, more false positive predictions will be introduced. In contrast, a higher threshold of anomaly score reduces the number of false positives but leads to lower recall. Generally, the threshold of anomaly score within the range of 0.9 to 0.95 is recommended.

We also evaluated the different threshold of read breakpoint ratio used for detecting error regions in isolate genomic assemblies. In a similar way, we set different threshold of read breakpoint ratio ranging from 0 to 0.6 with the threshold of anomaly score fixed as 0.95. The criteria of read breakpoint ratio >0.2 achieved the highest F1 score, and the recall would decrease if a higher threshold read breakpoint ratio was set.

## Parameter settings for tools

1、The MEGAHIT, IDBA_UD and metaSPAdes parameter settings used to obtain metagenomic assemblies

MEGAHIT was run with the "--k-list 21,29,39,59,79,99 --min-contig-len 1000" parameter settings.

IDBA_UD was run with the "--mink 20 --maxk 100 --step 20 --min_contig 1000" parameter settings.

metaSPAdes was run with the "-k 21, 29, 39, 59, 79, 99" parameter settings.

2、The BWA-MEM and samtools parameter settings used to generate BAM file

BWA-MEM was run with “-k 19 ” parameter settings.

samtools view was run with “-h -q 10 -m 50 -F 4” parameter settings to filter low quality mappings, and the alignment quality score cutoff ensures that the read is perfectly aligned.

samtools mpileup was run with “-C 50 -A -f” parameter settings to generate nucleotide variants information.

3、The parameter settings used for ScaffMatch to scaffold contigs assembled in isolate genomic datasets, ScaffMatch was run with the following parameters depending on the insert size of the dataset used

B.cereus dataset: “-i 600 -p fr -s 154”

R.sphaeroides datset: “-i 540 -p fr -s 65”

M.abscessus datset: “-i 200 -p fr -s 55”

V.cholerae dataset: “-i 200 -p fr -s 24”

## Evaluating metaMIC on contigs shorter than 5,000bp

In the main text, we mainly evaluated the performance of metaMIC on contigs with length longer than 5,000bp as approximately 70~80% of misassemblies happen in 5kb+ contigs (Fig. S26) and the probability of a contig misassembled increases as the contig becomes longer (see Figure S27). To explore the performance of metaMIC on contigs shorter than 5,000bp, we trained metaMIC on training datasets with contigs shorter than 5,000bp, then the trained model was used to predict misassemblies on contigs shorter than 5,000bp in CAMI1 datasets. As shown in Figure S28, metaMIC still performs best among all tools.

## Application to mock community

To demonstrate the usage of metaMIC on non-simulated metagenomic datasets, we made use of GIS20 mock community which were sequenced from mixture of 20 cultured organisms. GIS20 dataset has both complexity of real datasets but also the known ground-truth for evaluating accuracy of metagenomic assembly, and is available in the NCBI Sequence Read Archive [NCBI project ID: PRJEB29139] with a total number of 210,528,166 reads and 42.11G

bases. Microbial organisms in the mock community and reference genomes used for evaluation are listed in Table S10. Illumina paired-end reads from the dataset were assembled by MEGAHIT, and the accuracy of resulting contigs were evaluated by MetaQUAST through using 17 available reference genomes as gold standard. We evaluated both the accuracy of misassembled contig identification and misassembly breakpoint localization in misassembled contigs, and the performance of metaMIC on GIS20 dataset was shown in Figure S19.

# Supplementary Figures

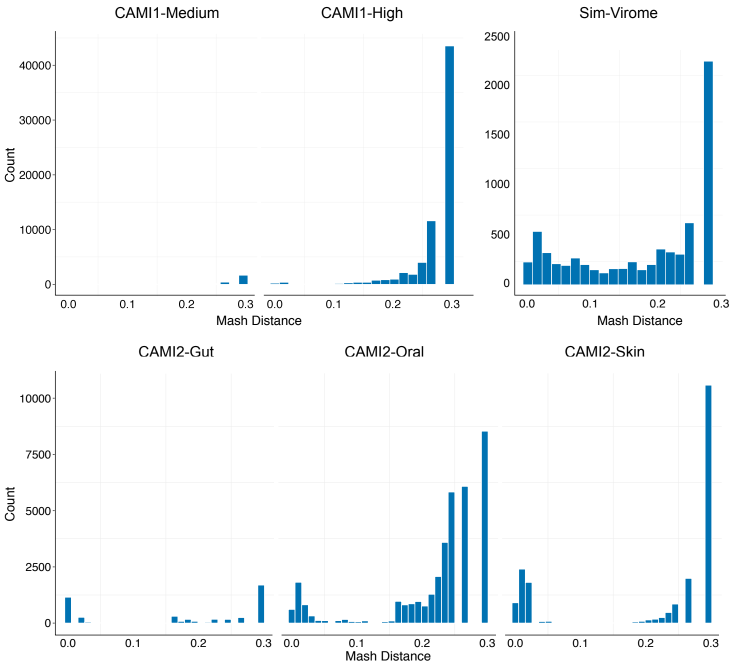


**Fig. S1** Mash distances of reference genomes used for medium-complexity (*CAMI1-Medium*) and high-complex communities (*CAMI2-High*), simulated virome dataset (*Sim-Virome*) and simulated microbial datasets from three different human body sites including gastrointestinal tract (*CAMI2-Gut*), skin (*CAMI2-Skin*) and oral cavity (*CAMI2-Oral*).

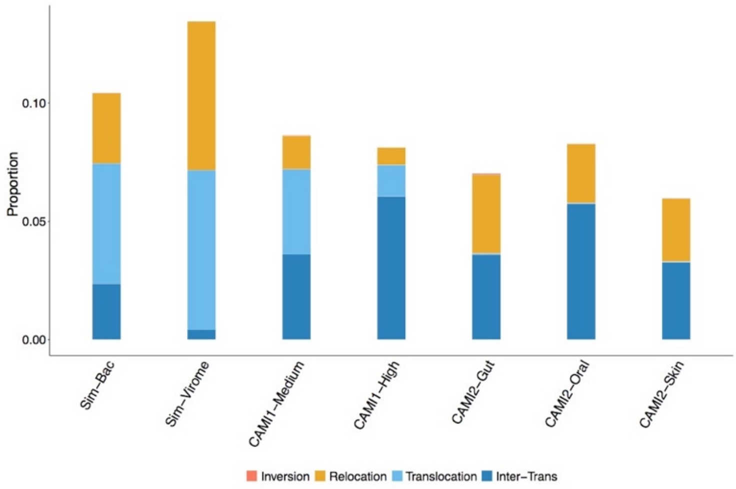


**Fig. S2** Distribution of misassembly types defined by metaQUAST on seven simulated datasets. One simulated bacterial metagenomics (*Sim-Bac*, Training datasets), one simulated virome dataset (*Sim-Virome*), and five simulated bacterial datasets from CAMI. Contigs from each dataset were all assembled by MEGAHIT. The label "Inter-Trans" stands for inter-genome translocation.

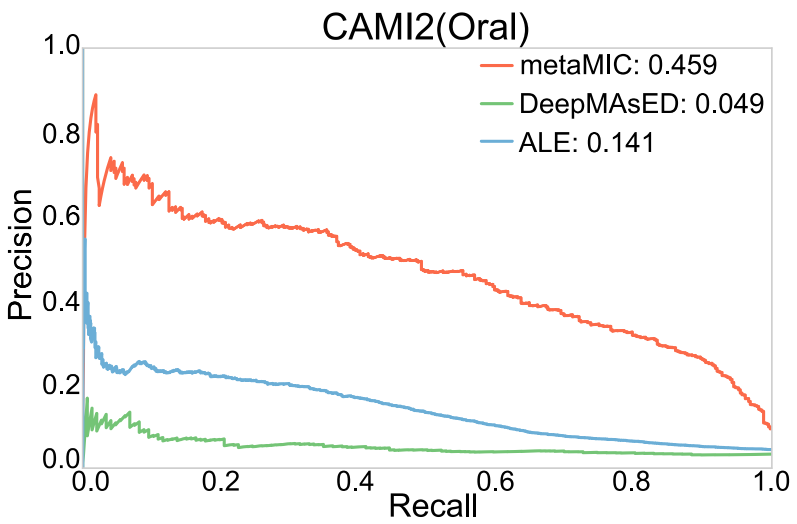


**Fig. S3** Comparison of metaMIC against ALE and DeepMAsED when identifying misassembled contigs in CAMI2-Oral. The contigs from this dataset were assembled by MEGAHIT.

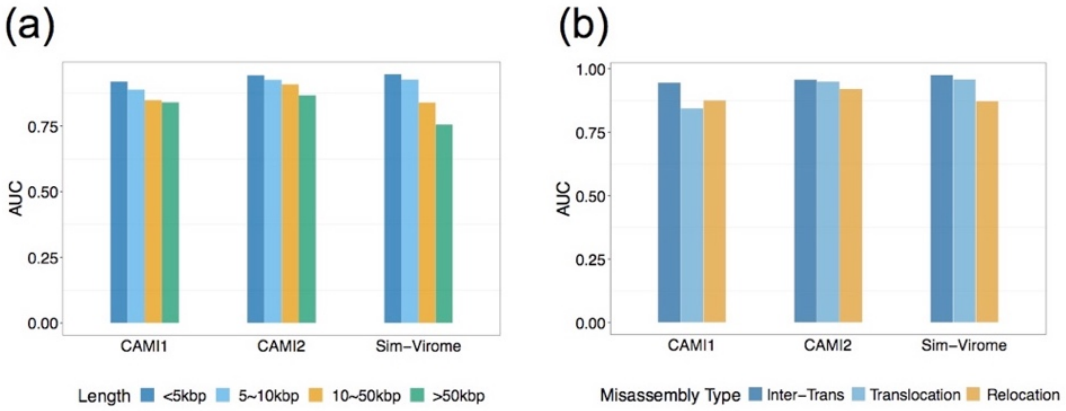


**Fig. S4 a, b** The impact of contig length (**a**) and misassembly type (**b**) on the performance of metaMIC. There is only few numbers of inversions, which is why there are not shown.

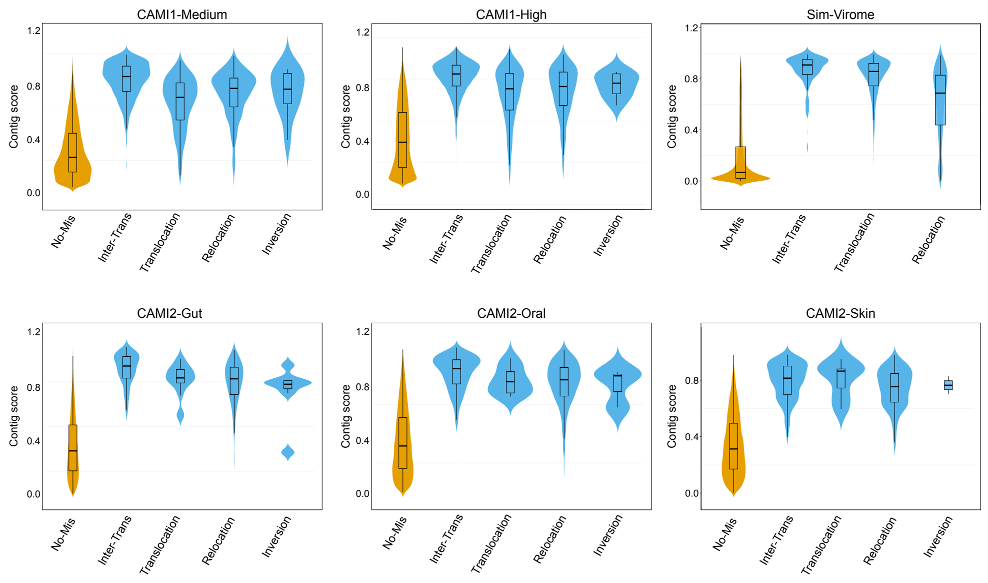


**Fig. S5** Distribution of metaMIC contig score for different misassembly types. Contigs from these datasets were all assembled by MEGAHIT. There is no inversion on Sim-Virome datasets, which is why there are not shown.

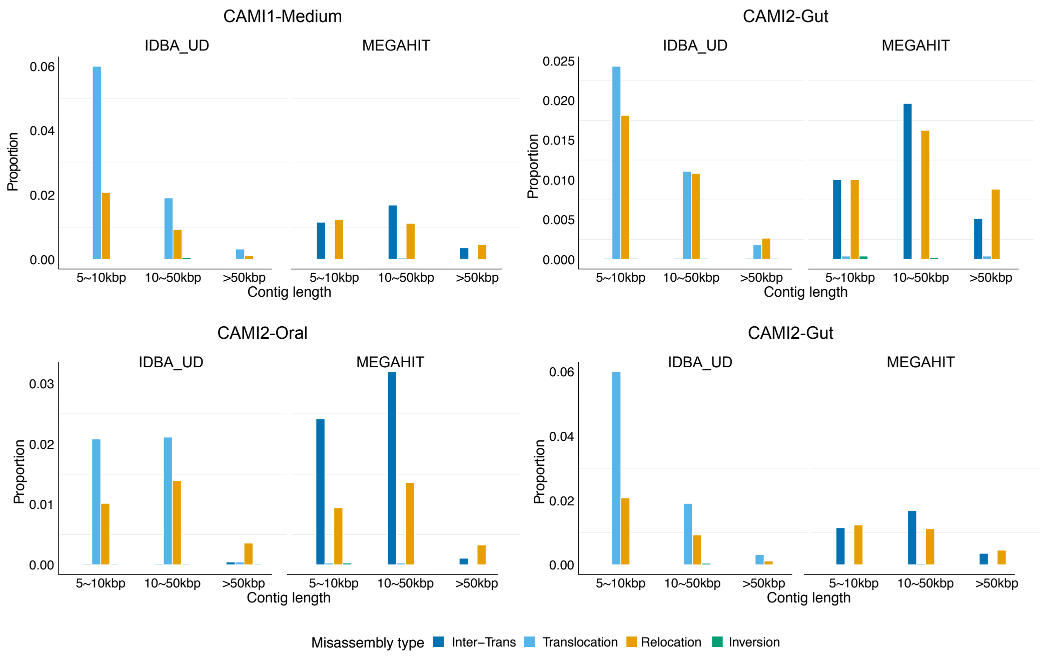


**Fig. S6** Distribution of misassembly types on different contig lengths and assemblers (IDBA_UD and MEGAHIT).

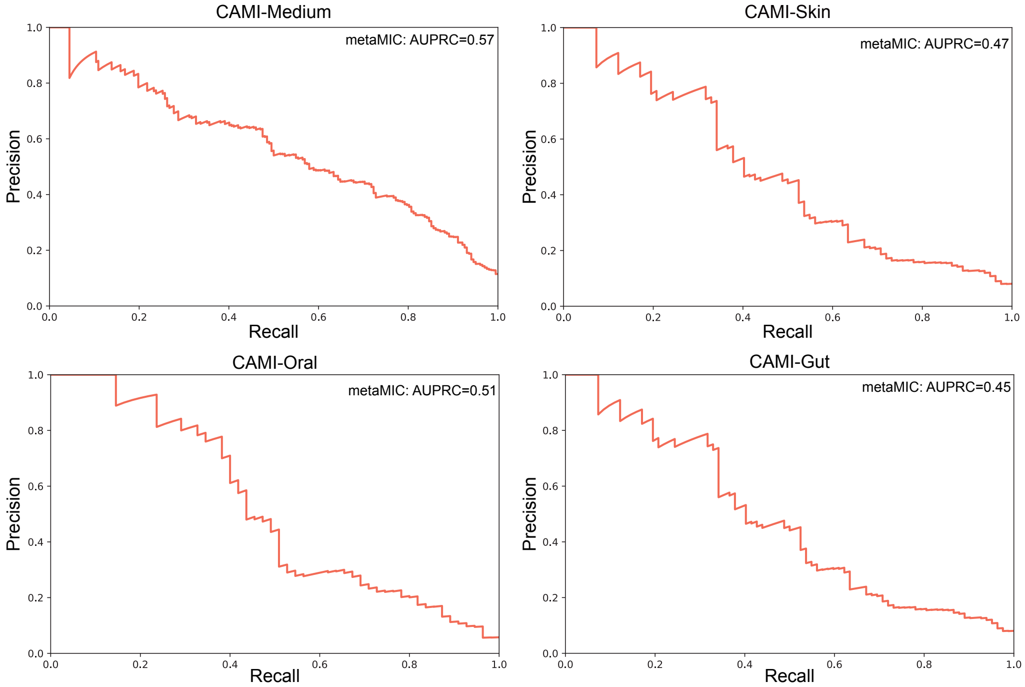


**Fig. S7** Precision-recall curves for identifying misassembled contigs in the CAMI datasets., where contigs from CAMI datasets were assembled by metaSPAdes. CAMI-High was excluded due to insufficient memory for metaSPAdes assembling.

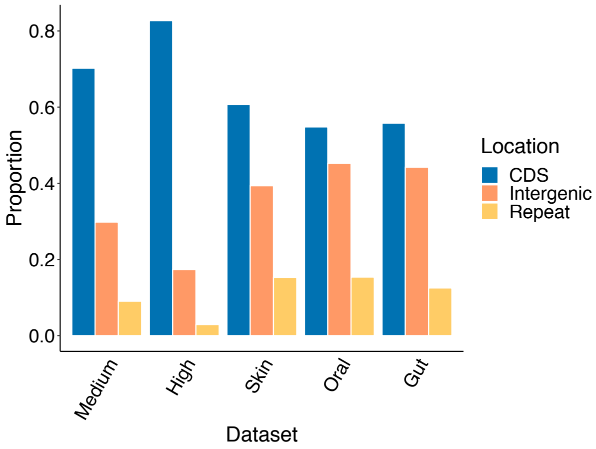


**Fig. S8** The proportion of misassembly breakpoints located in coding sequences (CDS), intergenic and repeat regions in the CAMI datasets. The CDS and intergenic regions were predicted by the Prodigal (v2.6.3) [51], whereas the repeat regions were determined by TRF (v4.07) [52] and RepeatScout (v1.0.6) [53]. All contigs were assembled by MEGAHIT.

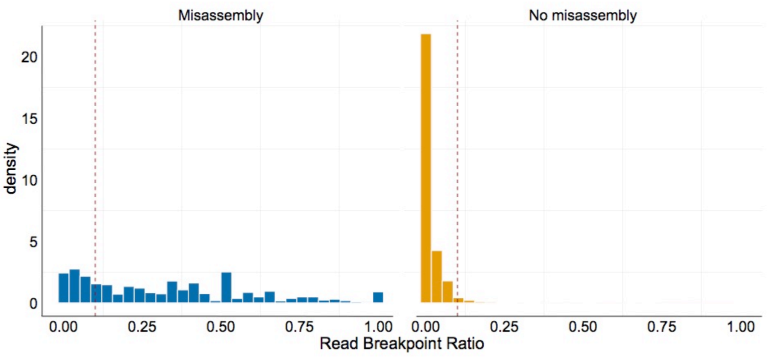


**Fig S9** Histogram of read breakpoint ratio on error regions containing misassembly breakpoints (Misassembly) and error-free regions (No misassembly) in the metagenomic assemblies.

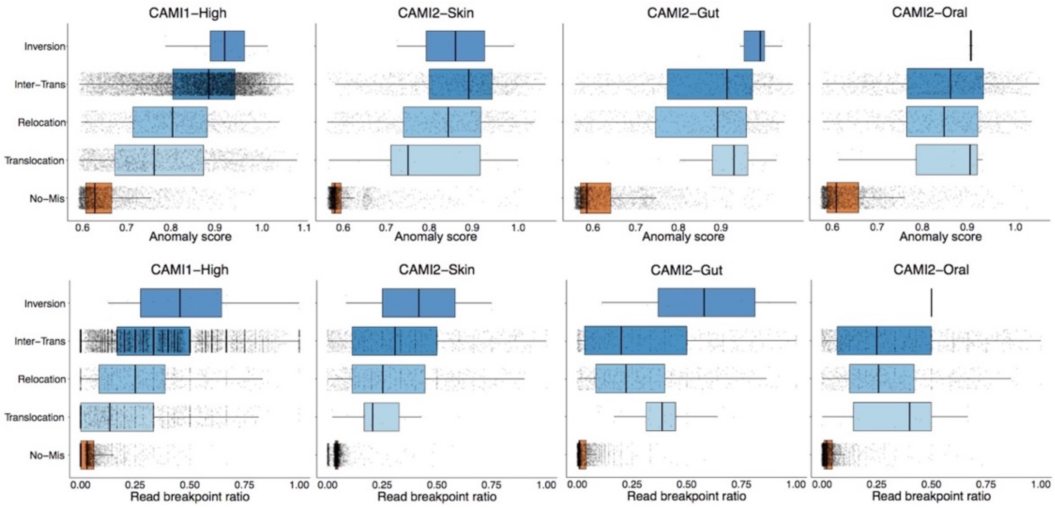


**Fig. S10** The distribution of anomaly scores (Top) and read breakpoint ratios (Bottom) of different misassembly types across contigs from CAMI datasets. All contigs from these datasets were assembled by MEGAHIT.

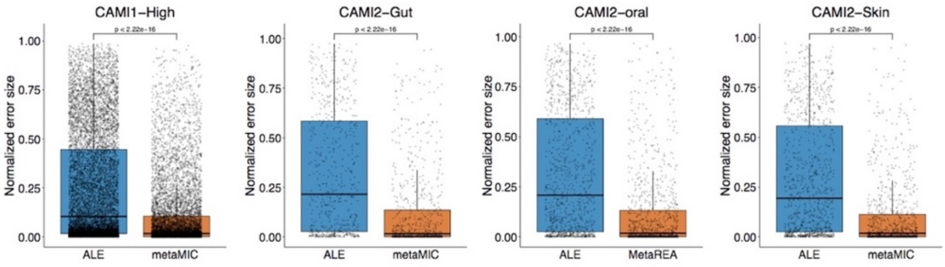


**Fig. S11** The distribution of normalized error size of misassembly breakpoints recognized by metaMIC and ALE on CAMI datasets. All contigs from these datasets were assembled by MEGAHIT.

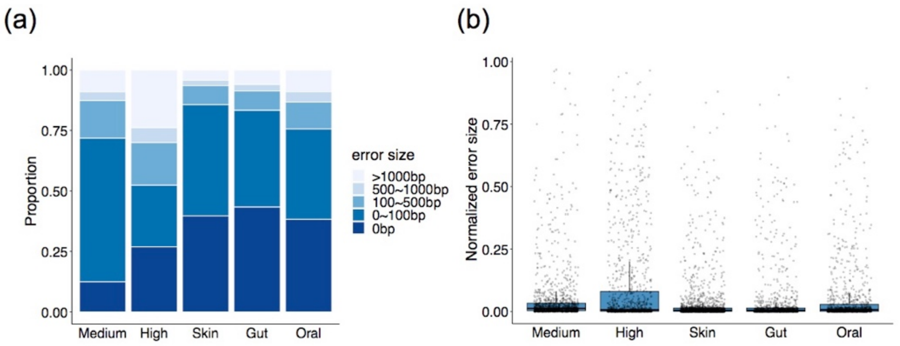


**Fig. S12** The performance of metaMIC in localizing misassembly breakpoints on CAMI datasets, where all contigs were assembled by IDBA_UD. **a, b** The distribution of error size (**a**) and normalized error size (**b**) of misassembly breakpoints recognized by metaMIC on CAMI datasets.

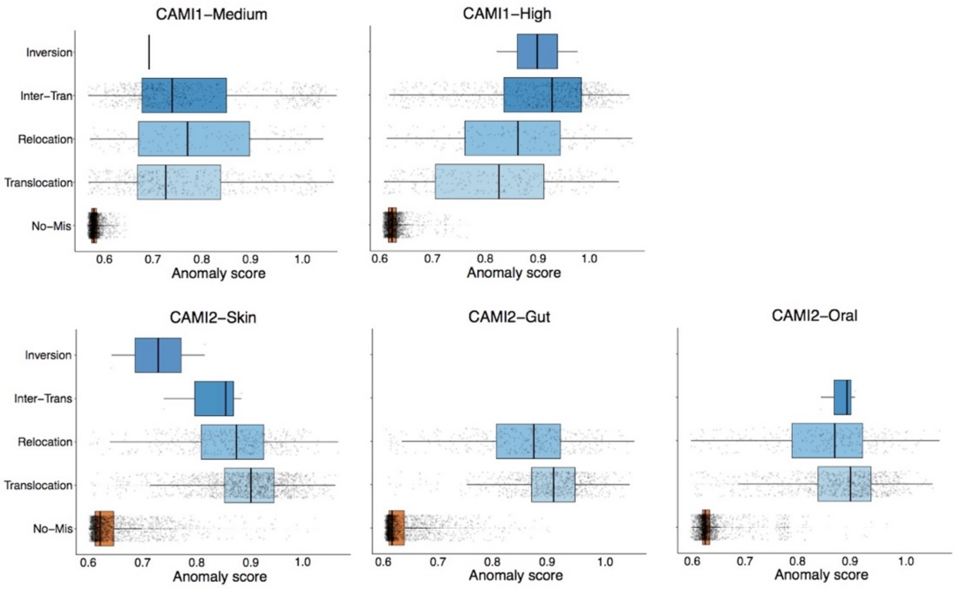


**Fig. S13** The distribution of anomaly scores of different misassembly types across contigs from CAMI datasets. All contigs from these datasets were assembled by IDBA_UD.

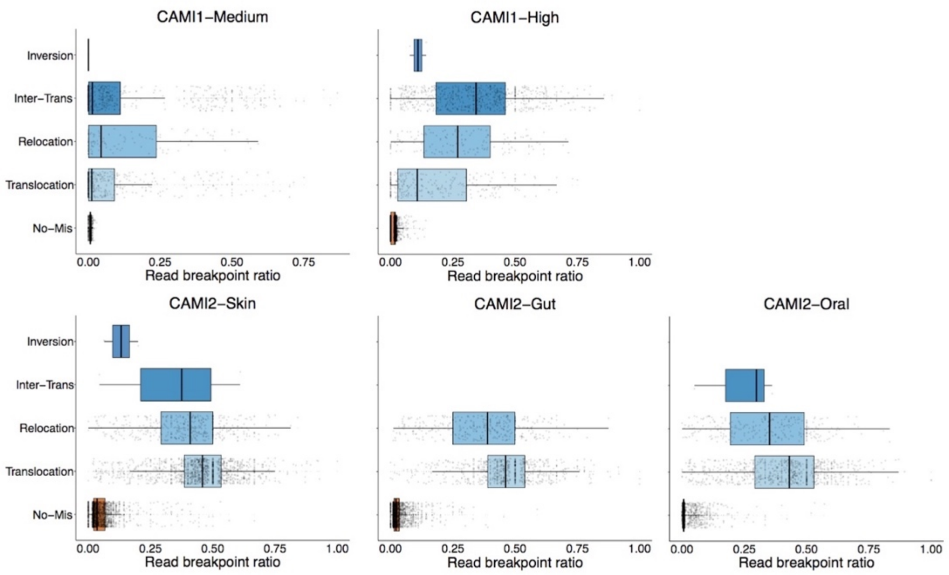


**Fig. S14** The distribution of read breakpoint ratio of different misassembly types across contigs from CAMI datasets. All contigs from these datasets were assembled by IDBA_UD.

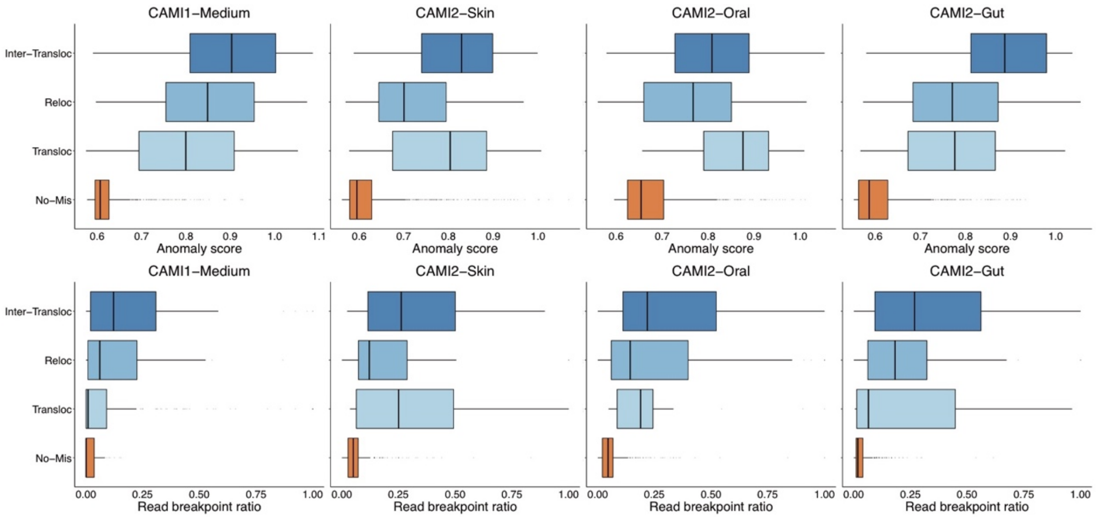


**Fig. S15** The distribution of anomaly scores (Top) and read breakpoint ratios (Bottom) of different misassembly types across contigs from CAMI datasets. All contigs from these datasets were assembled by metaSPAdes.

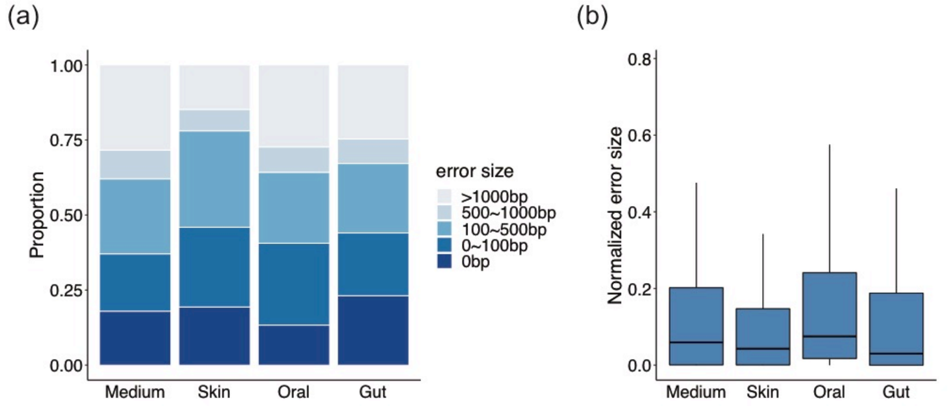


**Fig. S16** The performance of metaMIC in localizing misassembly breakpoints on CAMI datasets, where all contigs were assembled by metaSPAdes. **a, b** The distribution of error size (**a**) and normalized error size (**b**) of misassembly breakpoints recognized by metaMIC.

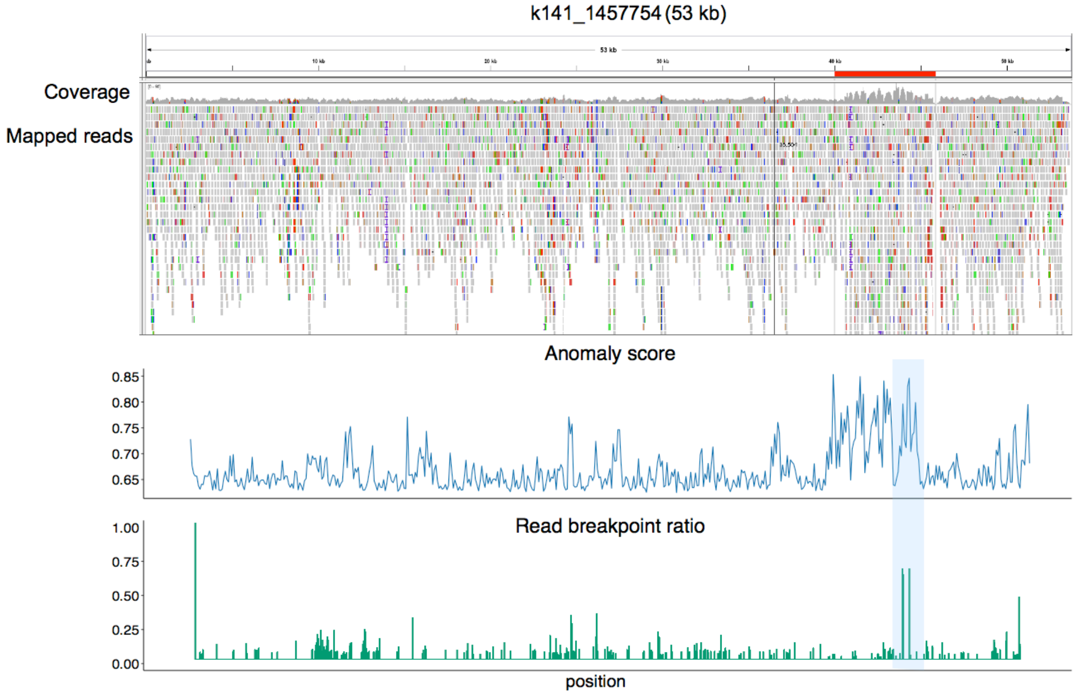


**Fig. S17** An example of a predicted misassembled contig "k141_1457754" assembled from combined rumen fluid and solid sample. The top plot shows a snapshot of Integrative Genomics Viewer for contig "k141_847840". The bottom plot shows the anomaly score (blue) and read breakpoint ratio (green) across contig "k141_1457743".

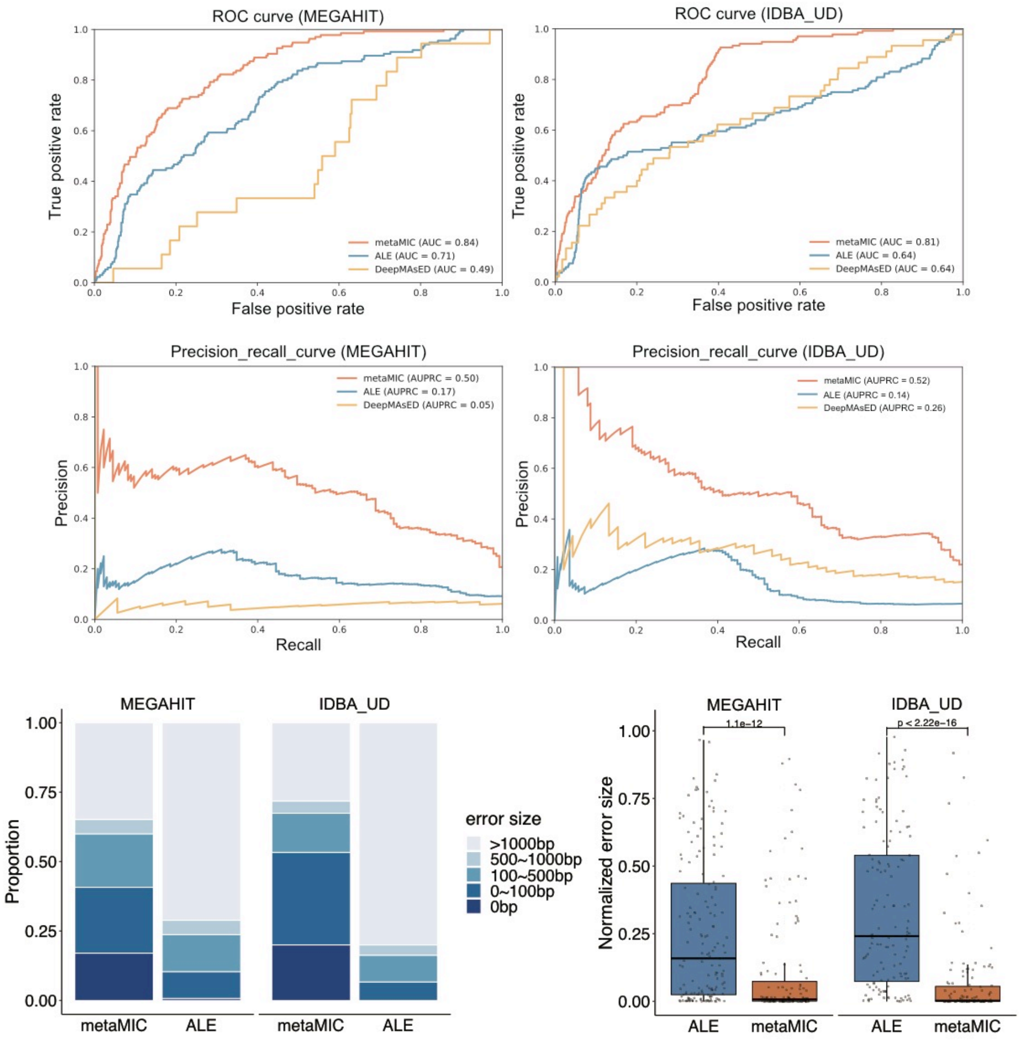


**Fig. S18** The performance of metaMIC for identifying misassemblies in the GIS20 datasets, where contigs were assembled by MEGAHIT and IDBA_UD, separately. The first two rows show benchmarking results of metaMIC against ALE and DeepMAsED for identifying misassembled contigs. The last row shows the distribution of error size (left) and normalized error size (right) of misassembly breakpoints recognized by metaMIC and ALE.

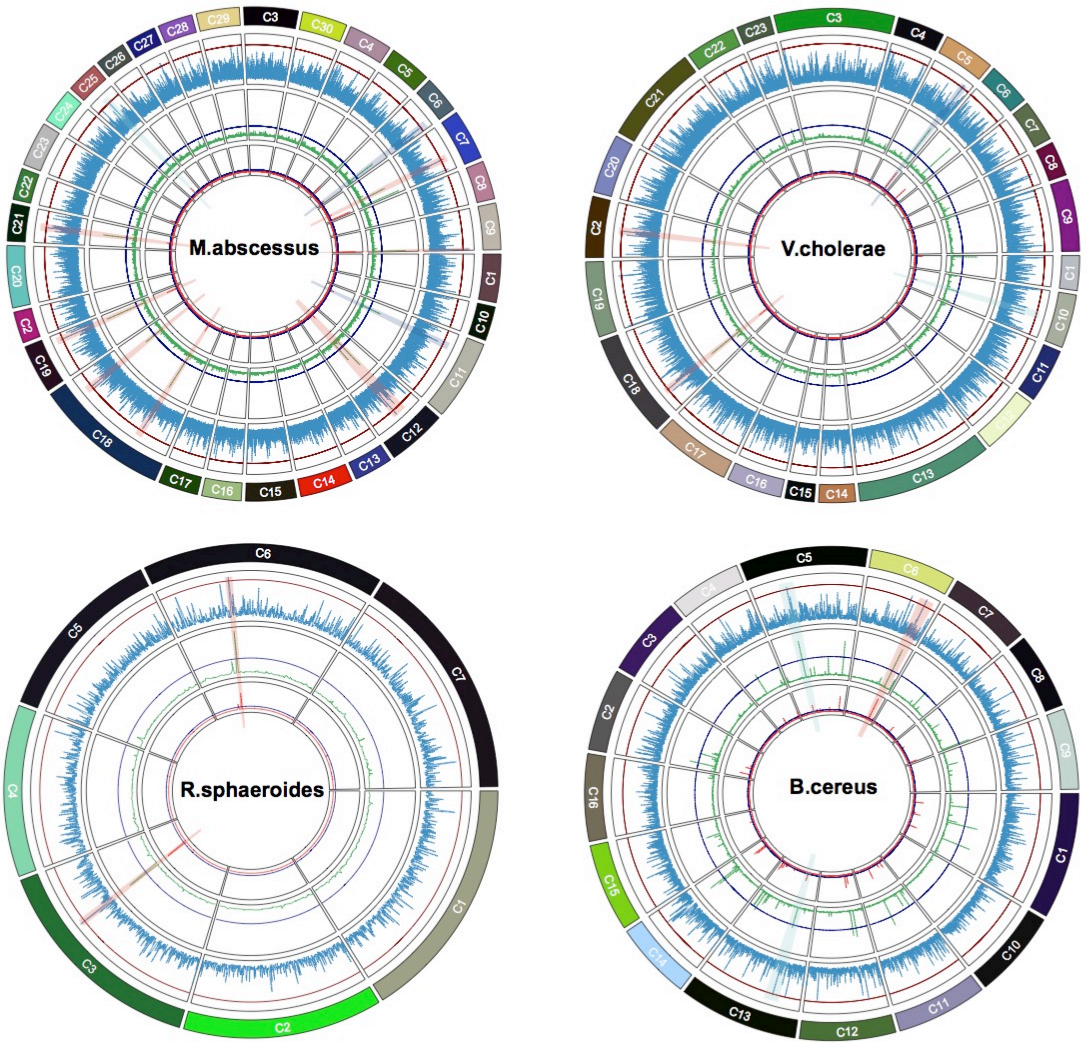


**Fig. S19** Visualization results of metaMIC on four real datasets from GAGE-B project. The outermost circle represents variable contigs assembled from datasets. The second circle (blue) and the third circle (green) shows anomaly scores and read breakpoint ratio across the assembled contigs. The innermost circle (red) shows the plot of read breakpoint counts across the assembled contigs. Only contigs with length >50,000bp were shown for clearness. Thresholds for anomaly score, read breakpoint ratio and read breakpoint count are 5, 0.1 and 0.95, respectively. The true misassemblies identified by metaMIC are marked as red bands. The true misassemblies that have not been identified by metaMIC are marked as purple bands, while the false predicted misassemblies are marked as blue bands.

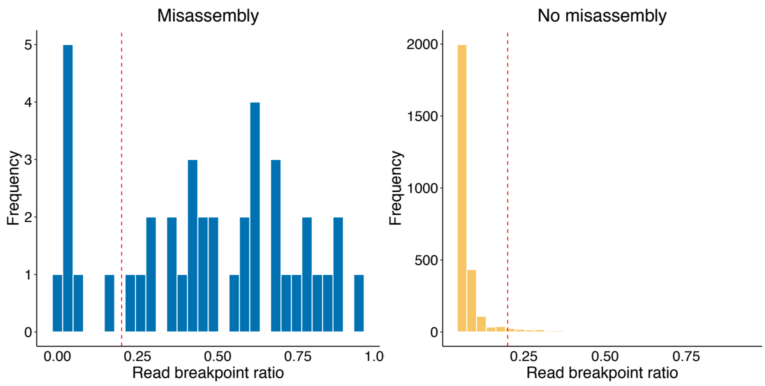


**Fig. S20** Histogram of read breakpoint ratio on error regions containing misassembly breakpoints (Misassembly) and error-free regions (No misassembly) in isolate genomic assemblies.

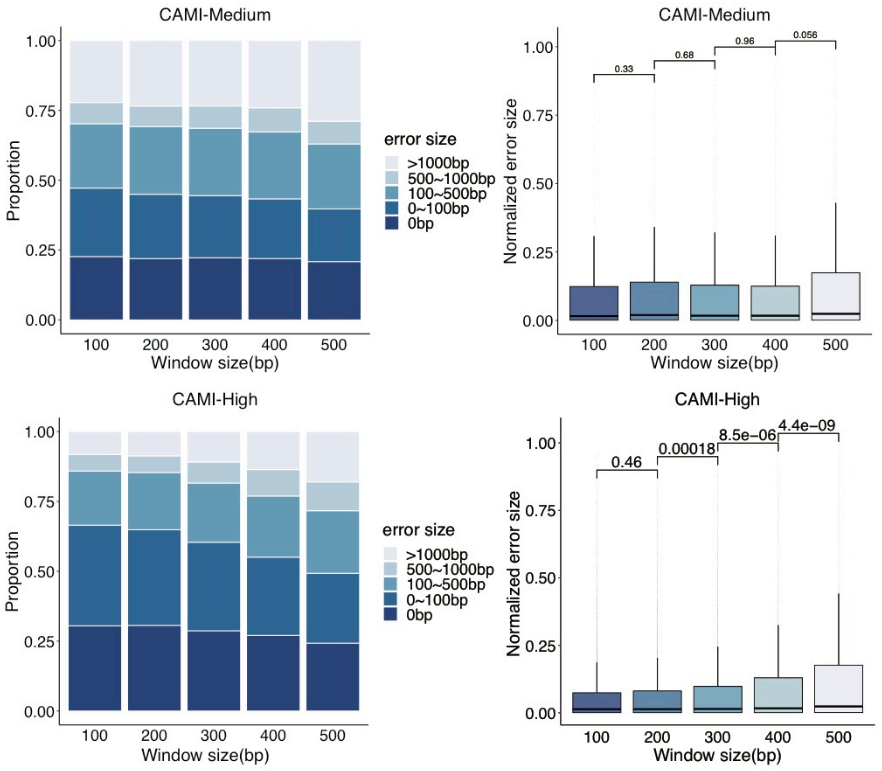


**Fig. S21** Selection of different size of sliding windows used to detect error regions in the CAMI-Medium (first row) and CAMI-High (second row) datasets.

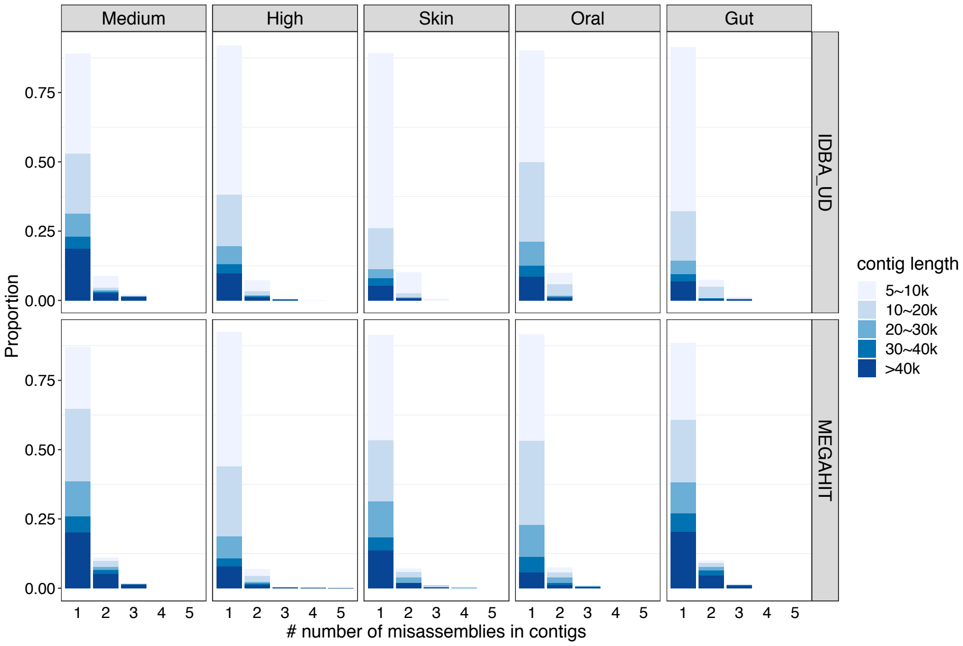


**Fig. S22** Distribution of the number of misassemblies contained wihtin each misassembled contig in the CAMI datasets, where contigs from these datasets were assembled by IDBA_UD and MEGAHIT, separately.

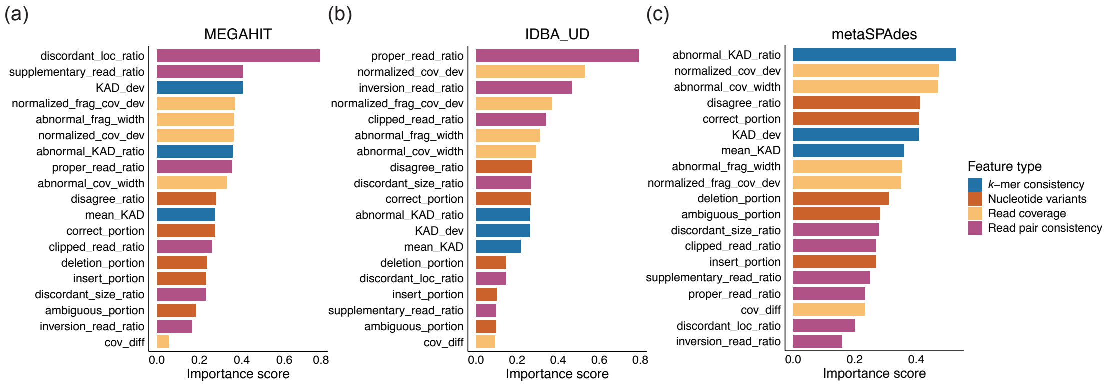


**Fig. S23** Ranking of feature importance of the random forest classifier trained on (a) MEGAHIT-specific training dataset, (b) IDBA_UD-specific training dataset and (c) metaSPAdes-specific training dataset, respectively.

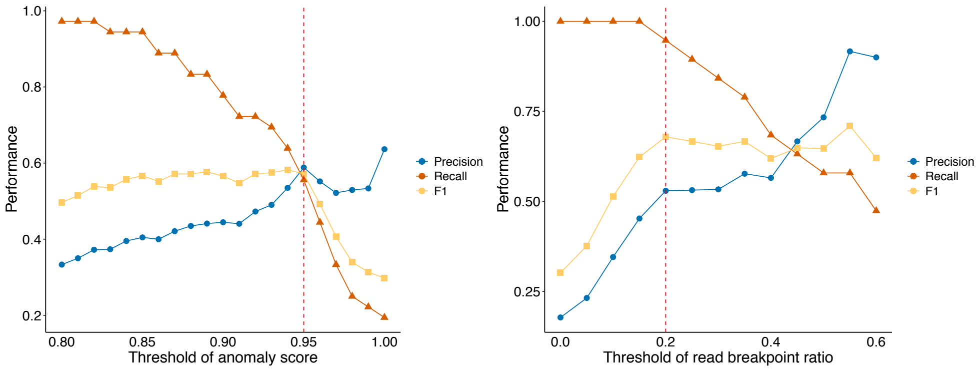


**Fig. S24** Selection of different threshold of anomaly score (left) and read breakpoint ratio (right) used to detect error regions in isolate genomic assemblies. Threshold of anomaly score was set 0.95, whereas threshold of read breakpoint ratio was set 0.2.

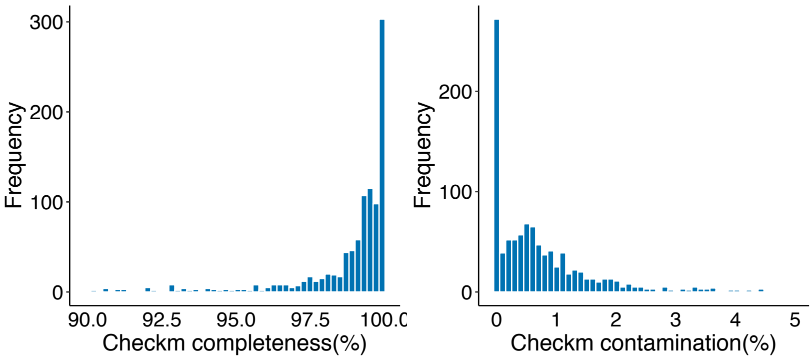


**Fig. S25** CheckM-estimated completeness and contamination of 1,000 bacterial genomes used for generating training datasets.

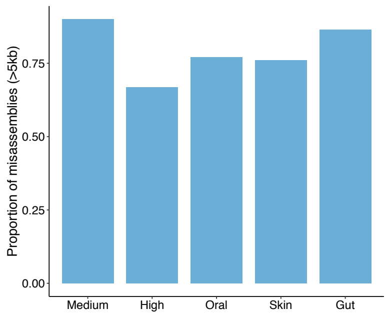


**Fig. S26** Proportion of extensive misassemblies occurred in contigs longer than 5,000bp. Contigs from the CAMI datasets were all assembled by MEGAHIT.

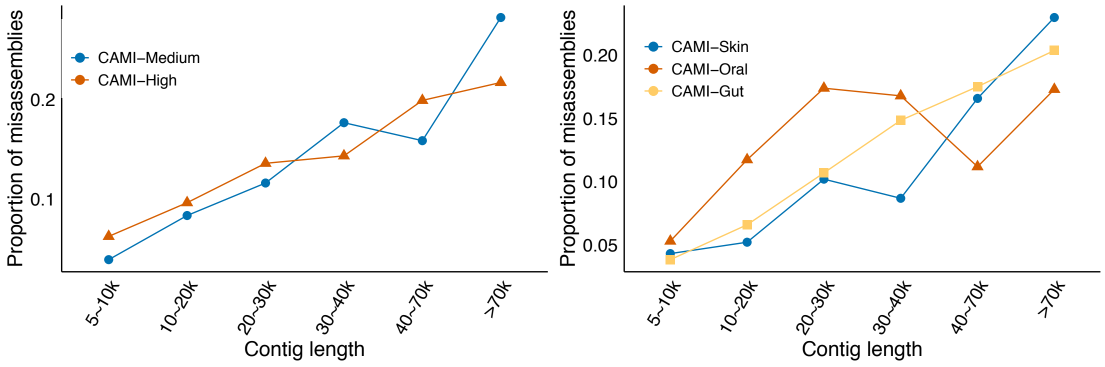


**Fig. 27** Proportion of misassembles on contigs grouped according to their length. Contig from these datasets were all assembled by MEGAHIT.

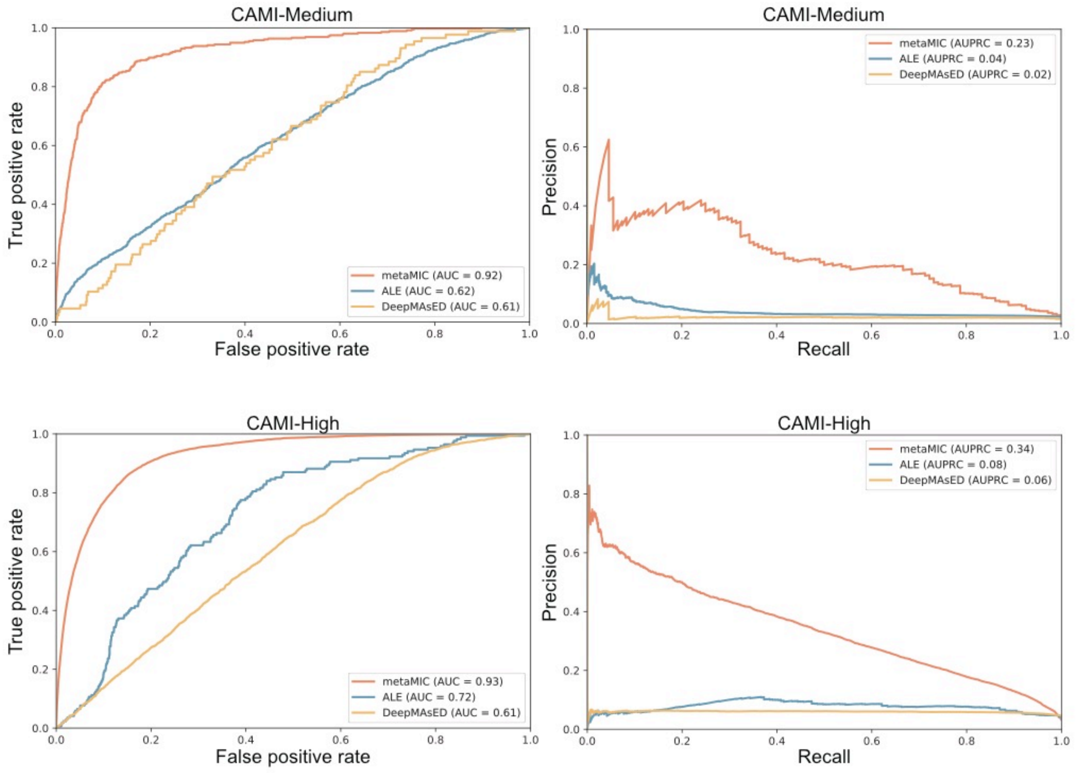


**Fig. S28** Comparison of metaMIC against ALE and DeepMAsED when identifying misassemblies in contigs shorter than 5,000bp from CAMI datasets. Contigs from these datasets were all assembled by MEGAHIT.

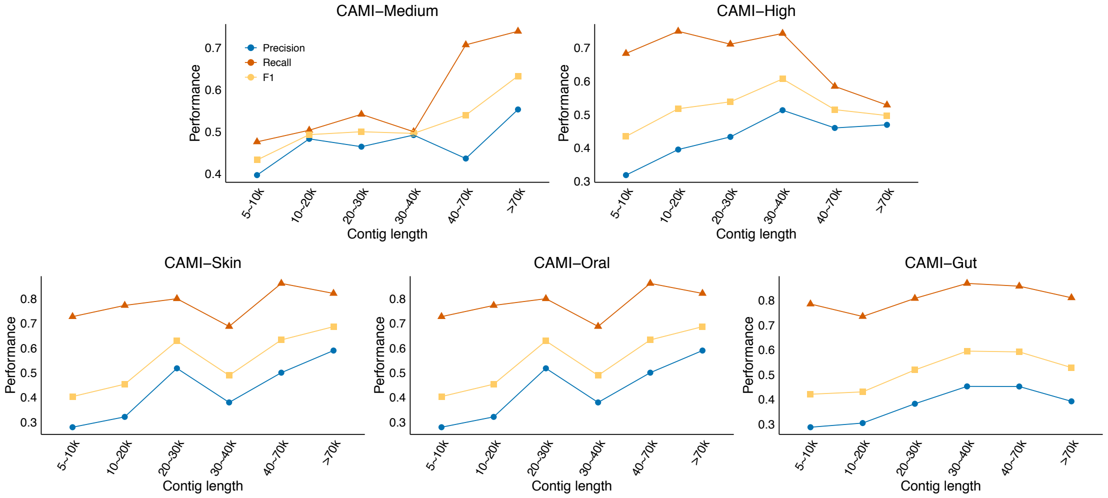


**Fig. S29** Performance of metaMIC on contigs grouped according to their length. The first low shows metaMIC performance on CAMI1 datasets, the second row shows the performance on CAIM2 datasets. Contigs from these datasets were assembled by MEGAHIT. Thresholds of metaMIC score for each dataset were set 0.6.

**References**


52. Hyatt D, Chen GL, Locascio PF, Land ML, Larimer FW, Hauser LJ. Prodigal: prokaryotic gene recognition and translation initiation site identification. BMC Bioinformatics. 2010;11:119.
53. Benson G. Tandem repeats finder: a program to analyze DNA sequences. Nucleic Acids Res. 1999;27:573-580.
54. Price AL, Jones NC, Pevzner PA. De novo identification of repeat families in large genomes. Bioinformatics. 2005;21 Suppl 1:i351-358.
